# Numerical Simulation of Hybrid Nanofluid Mixed Convection in a Lid-Driven Square Cavity with Magnetic Field Using High-Order Compact Scheme

**DOI:** 10.3390/nano11092250

**Published:** 2021-08-31

**Authors:** M. M. Rashidi, M. Sadri, M. A. Sheremet

**Affiliations:** 1 Institute of Fundamental and Frontier Sciences, University of Electronic Science and Technology of China, Chengdu 610054, China; mSadri@yahoo.com; 2 Faculty of Mechanical and Industrial Engineering, Quchan University of Technology, Quchan, Iran; 3 Laboratory on Convective Heat and Mass Transfer, Tomsk State University, 634050 Tomsk, Russia

**Keywords:** lid-driven square cavity, mixed convection, compact finite difference scheme, hybrid nanofluid, magnetic field

## Abstract

In this study, the energy transference of a hybrid Al_2_O_3_-Cu-H_2_O nanosuspension within a lid-driven heated square chamber is simulated. The domain is affected by a horizontal magnetic field. The vertical sidewalls are insulated and the horizontal borders of the chamber are held at different fixed temperatures. A fourth-order accuracy compact method is applied to work out the vorticity-stream function view of incompressible Oberbeck–Boussinesq equations. The method used is validated against previous numerical and experimental works and good agreement is shown. The flow patterns, Nusselt numbers, and velocity profiles are studied for different Richardson numbers, Hartmann numbers, and the solid volume fraction of hybrid nanoparticles. Flow field and heat convection are highly affected by the magnetic field and volume fraction of each type of nanoparticles in a hybrid nanofluid. The results show an improvement of heat transfer using nanoparticles. To achieve a higher heat transmission rate by using the hybrid nanofluid, flow parameters like Richardson number and Hartmann number should be considered.

## 1. Introduction

During past years, many efforts have been made to achieve the reasonable thermal efficiency of systems. Improvement of the heat transfer rate through a mixed convective flow [1] and adding nanoparticles are the part of these efforts [2]. The mixed convective circulation in a cavity with a heated lower wall has been investigated computationally by Moallemi and Jang [3]. They examined the influence of the Prandtl number on the rate of energy transference and flow dynamics. Iwatsu et al. [4] studied how heat was transferred inside a cavity with a temperature difference at the horizontal walls. In addition, in processes like casting and cooling of liquid metals, the magnetic field acts as an external force and affects the flow field and heat convection [5]. Many types of research have been studied to determine the significance of the hydromagnetic field on flow field and heat convection with various computational and analytical techniques. Chamkha [6] researched patterns of heat and flow by free convection of heat absorption and generation enclosures with a magnetic field. His results have shown that the magnetic strength strongly affects the heat convection and flow parameters of the chamber. Al-Salem et al. [7] studied the impact of the direction of a moving wall on magneto-hydrodynamic (MHD) mixed convection with different Grashof numbers, Hartmann numbers, and Reynolds numbers. They realized that flow field and heat convection are affected by the direction of lid movement and also empowering of the magnetic force causes a poor heat transfer. By applying a magnetic field, a Lorentz force in the opposite of the flow direction is generated. This magnetic force reduces the convective flow and heat transfer rate. The Lorentz force, which takes into account a body force with the Navier–Stokes equations, is a combination of electrical current and magnetic field. It should be noted that the interaction between the magnetic field and the flow depends on the fluid viscosity, conductivity, and flow characteristics [8]. In other words, when non-ferrofluids and non-ferromagnetic particles are used in a cavity, forces due to magnetization do not apply to the flow.

In some applications, such as magnetic storage media, magnetic sensors, and cooling systems, the presence of a magnetic field is unavoidable, so some researchers noticed that adding nanoparticles significantly enhances the heat transfer [9,10,11,12]. Balla et al. [9] considered the effects of different nanoparticles in an inclined square cavity, which was affected by the magnetic field. Mohebbi and Rashidi [10] proved that energy transference intensifies with growth of the volume fraction of Al_2_O_3_ particles in an L-shaped chamber. Ma et al. [11] investigated the nanofluid natural convection in a baffled U-shaped enclosure in the presence of a magnetic field. They found that the rate of heat transfer is suppressed by the magnetic field. The mixed convection heat transfer of nanofluid flow in a vertical channel with sinusoidal walls under a magnetic field effect was investigated numerically by Rashidi et al. [12].

Another type of nanofluid that has recently received attention, is the hybrid nanofluid. Simultaneous combinations of a metallic nanoparticle with its non-metallic type increases the thermal conductivity as well as stability of the nanofluid [13]. In this way, the properties of two or three nanoparticles can be used. For example, metal nanoparticles have high thermal conductivity but can cause a chemical reaction in the fluid, while non-metallic nanoparticles have high stability despite low thermal conductivity [13]. Until now, many research studies have been done in the field of hybrid nanofluids. Moghadassi et al. [14] compared the specifications of Al_2_O_3_-H_2_O and Al_2_O_3_-Cu-H_2_O. They ascertained that the convective energy transference is far higher for the hybrid nanofluid. The heat conductivity and viscosity of Al_2_O_3_-Cu-H_2_O hybrid nanosuspension in a tube were analyzed by Suresh et al. [15]. They showed that energy transference is raised when the hybrid nanofluid is applied. In addition, Suresh et al. [16] studied the laminar flow in a heated tube filled with Al_2_O_3_-Cu-H_2_O hybrid nanofluid experimentally, and showed that the Nusselt number is increased in a hybrid nanofluid in comparison with pure water. Ghalambaz et al. [17] investigated an Ag-MgO/water hybrid nanofluid inside a square cavity. The effects of variation of the main parameters, such as the volume fraction of the nanoparticles and the Rayleigh number, were studied. The effect on the entropy production and MHD convection of the hybrid nanofluid Al_2_O_3_-Cu in a porous square enclosure was studied numerically by Abdel-Nour et al. [18]. They found that convective heat transfer becomes stronger with the enhancement of the Rayleigh number while it detracts with the rise in Hartmann number.

High-order mathematical simulation of incompressible Navier–Stokes equations has been performed by various researchers [19,20,21,22]. Garmann [21] has explored a sixth-order compact differencing method for solving incompressible flows such as steady lid-driven cavities and fluid flow around a cylinder. It was found that the presented method provided high accuracy solutions on coarse grids. In the current study, flow patterns and heat convection through a hybrid nanofluid are numerically studied. The energy equation and the vorticity-stream function formulation are computed by the high-order compact scheme. The influence of Richardson numbers, Hartmann numbers, and hybrid nano-sized particle concentration on the flow are studied comprehensively. The current research is arranged as follows. Section 2 expresses the governing equations. The numerical methodology is explained in Section 3. Section 4 gives the results of the selected problem. Finally, the conclusions are presented in Section 5.

## 2. Governing Equations

The continuity, momentum, and energy equations with thermal buoyancy and magnetic field are as follows [23]:

(1) $$\frac{\partial u}{\partial x}+\frac{\partial v}{\partial y}=0$$

(2) $$\frac{\partial u}{\partial t}+u\frac{\partial u}{\partial x}+v\frac{\partial u}{\partial y}=-\frac{1}{\rho_{hnf}}\frac{\partial p}{\partial x}+\frac{\mu_{hnf}}{\rho_{hnf}}\left(\frac{\partial^{2}u}{\partial x^{2}}+\frac{\partial^{2}u}{\partial y^{2}}\right)$$

(3) $$\frac{\partial v}{\partial t}+u\frac{\partial v}{\partial x}+v\frac{\partial v}{\partial y}=-\frac{1}{\rho_{hnf}}\frac{\partial p}{\partial y}+\frac{\mu_{hnf}}{\rho_{hnf}}\left(\frac{\partial^{2}v}{\partial x^{2}}+\frac{\partial^{2}v}{\partial y^{2}}\right)-\frac{\sigma_{hnf}B_{0}^{2}}{\rho_{hnf}}v+\frac{{\left(\rho \beta \right)}_{hnf}}{\rho_{hnf}}g\left(T-T_{c}\right)$$

(4) $$\frac{\partial T}{\partial t}+u\frac{\partial T}{\partial x}+v\frac{\partial T}{\partial y}=\alpha_{hnf}\left(\frac{\partial^{2}T}{\partial x^{2}}+\frac{\partial^{2}T}{\partial y^{2}}\right)$$

where *u* and *v* are the fluid velocity along *x*- and *y*-axes, *p* is the pressure, ρ is the density, *T* is the temperature, μ is the viscosity, α is the heat diffusivity, β is the coefficient of volumetric heat expansion, *B*_0_ is the magnitude of the applied magnetic force, and σ is the electrical conductivity. Note that subscript *hnf* refers to the hybrid nanofluid properties.

By nondimensionalizing Equations (1)–(4) with the following non-dimensional quantities $U=\frac{u}{U_{0}},V=\frac{v}{U_{0}},X=\frac{x}{H},Y=\frac{y}{H},P=\frac{p}{\rho_{f}U_{0}^{2}},\theta =\frac{T-T_{c}}{T_{h}-T_{c}},\tau =\frac{tU_{0}}{H}$ and taking the velocity components employing the dimensionless stream function ψ, defined as $U=\frac{\partial \psi }{\partial Y},V=-\frac{\partial \psi }{\partial X}$, and the dimensionless vorticity $\omega =\frac{\partial V}{\partial X}-\frac{\partial U}{\partial Y}$, Equations (1)–(4) are transformed into

(5) $$\frac{\partial^{2}\psi }{\partial X^{2}}+\frac{\partial^{2}\psi }{\partial Y^{2}}=-\omega$$

(6) ∂ω∂τ+∂ψ∂Y∂ω∂X−∂ψ∂X∂ω∂Y=1Reμhnfμfρfρhnf∇2ω+GrRe2ρβhnfρhnfβf∂θ∂X+σhnfσfρfρhnfHa2Re∂2ψ∂X2

(7) ∂θ∂τ+∂ψ∂Y∂θ∂X−∂ψ∂X∂θ∂Y=1RePrαhnfαf∂2θ∂X2+∂2θ∂Y2

where subscript *f* denotes the base fluid properties.

The dimensionless parameters used are Prandtl number $\left(\mathrm{Pr}=\mu_{f}/\left(\rho_{f}\alpha_{f}\right)\right)$, Reynolds number Re=ρfU0H/μf, Grashof number $\left(Gr=g\beta_{f}\rho_{f}^{2}H^{3}\left(T_{h}-T_{c}\right)/\mu_{f}^{2}\right)$, Richardson number Ri=Gr/Re2, and Hartmann number $\left(Ha=B_{0}H\sqrt{\sigma_{f}/\mu_{f}}\right)$.

In the above Equations (5)–(7), the hybrid Al_2_O_3_-Cu-H_2_O hybrid nanosuspension density, specific heat, thermal expansion, and thermal diffusivity are given by [13]:

(8) $$\rho_{hnf}=\phi_{{\mathrm{Al}}_{2}O_{3}}\rho_{{\mathrm{Al}}_{2}O_{3}}+\phi_{\mathrm{Cu}}\rho_{\mathrm{Cu}}+\left(1-\phi \right)\rho_{f}$$

(9) $${\left(\rho c\right)}_{hnf}=\phi_{{\mathrm{Al}}_{2}O_{3}}{\left(\rho c\right)}_{{\mathrm{Al}}_{2}O_{3}}+\phi_{\mathrm{Cu}}{\left(\rho c\right)}_{\mathrm{Cu}}+\left(1-\phi \right){\left(\rho c\right)}_{f}$$

(10) $${\left(\rho \beta \right)}_{hnf}=\phi_{{\mathrm{Al}}_{2}O_{3}}{\left(\rho \beta \right)}_{{\mathrm{Al}}_{2}O_{3}}+\phi_{\mathrm{Cu}}{\left(\rho \beta \right)}_{\mathrm{Cu}}+\left(1-\phi \right){\left(\rho \beta \right)}_{f}$$

where ϕ is the nanoparticle volume fraction $\left(\phi =\phi_{{\mathrm{Al}}_{2}O_{3}}+\phi_{\mathrm{Cu}}\right)$

(11) $$\alpha_{hnf}=\frac{k_{hnf}}{{\left(\rho c\right)}_{hnf}}$$

The thermal conductivity of the hybrid nanosuspension *k_hnf_* and the electrical conductivity of the hybrid nanosuspension σ*_hnf_* are calculated by [24] as follows:

(12) $$\frac{k_{hnf}}{k_{f}}=\frac{\frac{\phi_{{\mathrm{Al}}_{2}O_{3}}k_{{\mathrm{Al}}_{2}O_{3}}+\phi_{\mathrm{Cu}}k_{\mathrm{Cu}}}{\phi }+2k_{f}+2\left(\phi_{{\mathrm{Al}}_{2}O_{3}}k_{{\mathrm{Al}}_{2}O_{3}}+\phi_{\mathrm{Cu}}k_{\mathrm{Cu}}\right)-2\phi k_{f}}{\frac{\phi_{{\mathrm{Al}}_{2}O_{3}}k_{{\mathrm{Al}}_{2}O_{3}}+\phi_{\mathrm{Cu}}k_{\mathrm{Cu}}}{\phi }+2k_{f}-\left(\phi_{{\mathrm{Al}}_{2}O_{3}}k_{{\mathrm{Al}}_{2}O_{3}}+\phi_{\mathrm{Cu}}k_{\mathrm{Cu}}\right)+\phi k_{f}}$$

(13) $$\frac{\sigma_{hnf}}{\sigma_{f}}=1+3\frac{\frac{\phi_{{\mathrm{Al}}_{2}O_{3}}\sigma_{{\mathrm{Al}}_{2}O_{3}}+\phi_{\mathrm{Cu}}\sigma_{\mathrm{Cu}}}{\sigma_{f}}-\phi }{\left(\frac{\phi_{{\mathrm{Al}}_{2}O_{3}}\sigma_{{\mathrm{Al}}_{2}O_{3}}+\phi_{\mathrm{Cu}}\sigma_{\mathrm{Cu}}}{\phi \sigma_{f}}+2\right)-\left(\frac{\phi_{{\mathrm{Al}}_{2}O_{3}}\sigma_{{\mathrm{Al}}_{2}O_{3}}+\phi_{\mathrm{Cu}}\sigma_{\mathrm{Cu}}}{\sigma_{f}}-\phi \right)}$$

The viscosity of the hybrid nanosuspension is calculated by the Brinkman model [25]:

(14) $$\mu_{hnf}=\mu_{f}\frac{1}{{\left(1-\phi \right)}^{2.5}}$$

## 3. Numerical Method

The derivatives in Equations (6) and (7) are calculated by the three-point fourth-order compact technique by the following tridiagonal system of equations [26]:

(15) $$\frac{1}{4}{\varphi^{\prime }}_{i-1}+{\varphi^{\prime }}_{i}+\frac{1}{4}{\varphi^{\prime }}_{i+1}=3\frac{\varphi_{i+1}-\varphi_{i-1}}{4h}+o\left(h^{4}\right)$$

(16) $$\frac{1}{10}{\varphi^{\prime \prime }}_{i-1}+{\varphi^{\prime \prime }}_{i}+\frac{1}{10}{\varphi^{\prime \prime }}_{i+1}=12\frac{\varphi_{i-1}-2\varphi_{i}+\varphi_{i+1}}{10h^{2}}+o\left(h^{4}\right)$$

where $\varphi^{\prime }$ and $\varphi^{\prime \prime }$ are the first and second derivatives of any variable φ. The following fourth-order difference approximation is applied for the numerical simulation of Equation (5) [27]:

(17) $$\begin{array}{l} \frac{2}{h^{2}}\left\{\left(\psi_{i+1,j+1}+\psi_{i-1,j+1}+\psi_{i+1,j-1}+\psi_{i-1,j-1}-20\psi_{i,j}\right)+4\left(\psi_{i+1,j}+\psi_{i,j+1}+\psi_{i-1,j}+\psi_{i,j-1}\right)\right\}=\\ 8\omega_{i,j}+\omega_{i-1,j}+\omega_{i,j-1}+\omega_{i+1,j}+\omega_{i,j+1} \end{array}$$

Equation (17) is solved by using the under-relaxation technique.

The stream function on the boundary is set as zero and the Neumann boundary restrictions for θ are considered. Note that the vorticity is not determined on the boundary, so the numerical boundary of the vorticity needs to be presented. By solving Equation (5) on the wall, the following fourth-order discretization is obtained for the vorticity magnitude on the boundaries [28]:

(18) $$h\left(6\omega_{1}+4\omega_{2}-\omega_{3}\right)/21=\left(15\psi_{1}-16\psi_{2}+\psi_{3}\right)/\left(14h\right)\pm V_{w}$$

where *V_w_* is the tangential wall velocity.

The convergence criterion is specified as follows:

(19) $$\frac{\sum \left|\varphi_{i,j}^{n+1}-\varphi_{i,j}^{n}\right|}{\sum \left|\varphi_{i,j}^{n+1}\right|}\leq 10^{-7}$$

where *n* is the iteration number. Note that in this problem, the iteration continues until all three field variables ω, ψ, and θ reach the convergence criterion.

As regards the Nusselt number, this can describe the heat convection specifications. Note that the average *Nu* on the top line is computed by the following equation:

(20) $$Nu_{ave.}=-\frac{k_{hnf}}{k_{f}}\int_{0}^{1}\frac{\partial \theta }{\partial Y}dX$$

## 4. Results and Discussion

In this section, a mixed convective motion with the applied Lorentz force is numerically simulated. The domain is a square chamber saturated with a hybrid Al_2_O_3_-Cu-H_2_O nanosuspension. A high-order in-house computational code was generated by the authors and was validated against numerical results that are available in the literature.

### 4.1. Problem Description and Boundary Conditions

**Figure 1:**
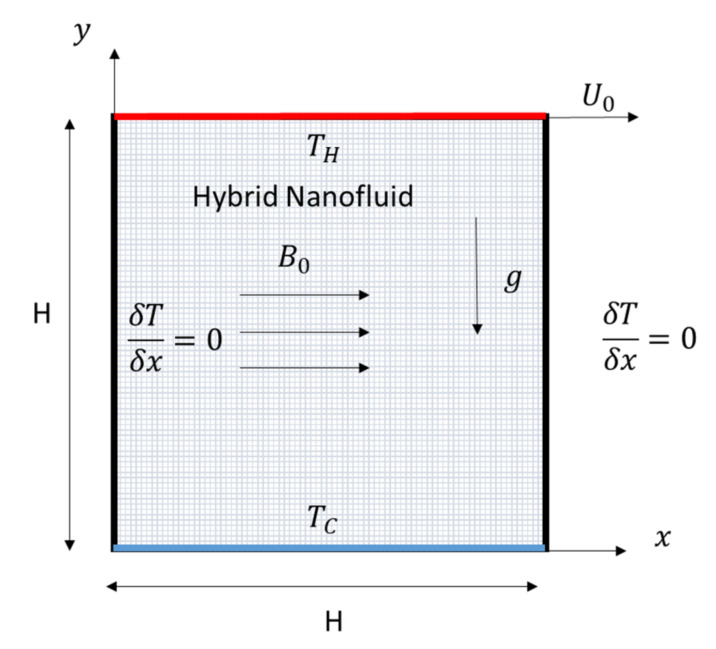
The physical domain of the square cavity.

The square cavity with constant different temperatures at the horizontal walls is displayed in Figure 1. The side boundaries are thermally insulated and the domain is affected by a uniform and horizontal magnetic force of strength *B*_0_. The top lid is moving to the right with velocity *U*_0_ and the other three boundaries are motionless. The properties of water as the host liquid, and the Al_2_O_3_ and Cu nano-sized particles are shown in Table 1.

### 4.2. Validation

**Figure 2:**
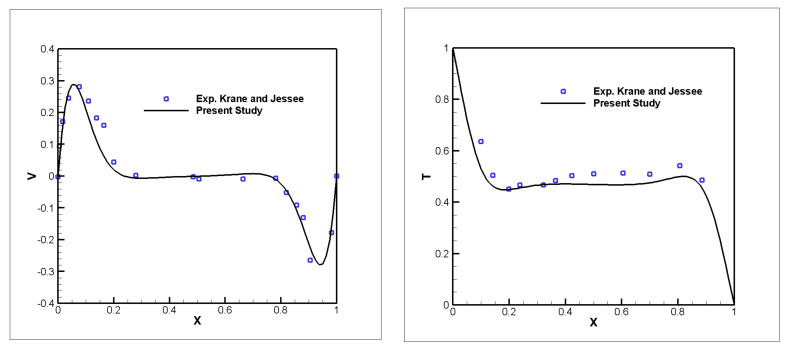
Comparison of temperature and vertical velocity profile between the present results and the experimental results by Krane and Jessee [29] (*Ra* = 1.89 × 10^5^).

To validate the proposed approach, the results were compared with the available experimental and numerical data from [23,29]. The experimental results of Krane and Jessee [29] for natural convection in a cavity filled with air are shown in Figure 2. It can be seen from the comparison that the current solution is in good agreement with the experimental data.

**Figure 3:**
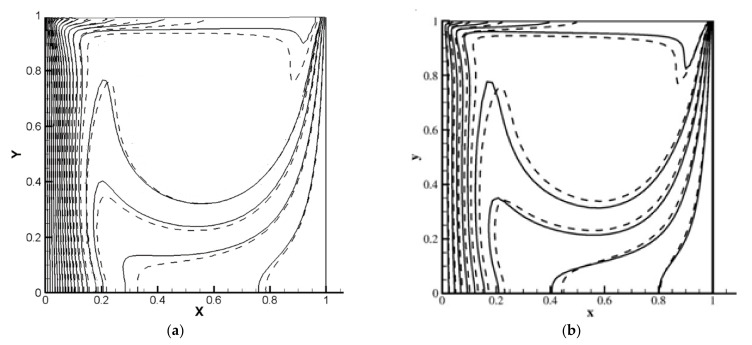
Isotherms for *Re* = 100 and *Ra* = 1.47 × 10^4^: φ = 0 ––––, φ = 0.05 -------. (**a**) Current study; (**b**) Talebi et al. [23].

**Figure 4:**
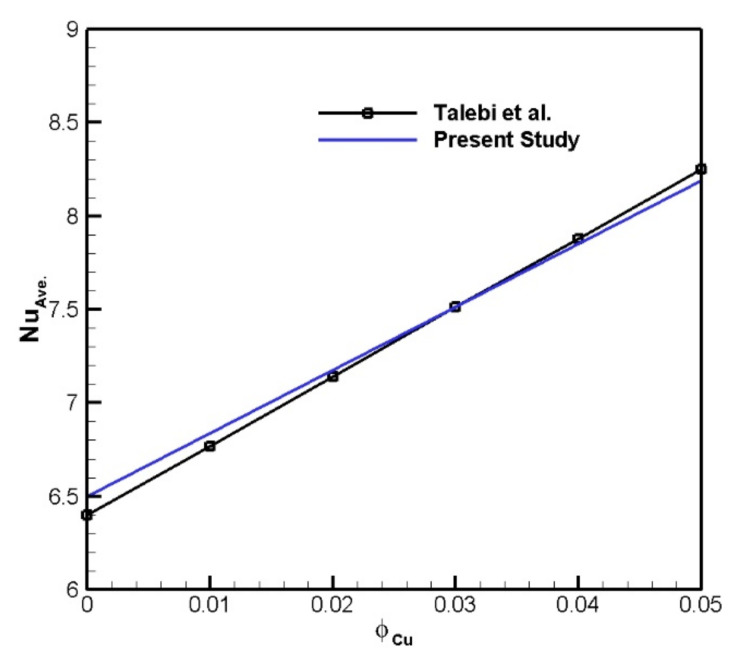
Average *Nu* versus volume fraction of Cu nanoparticles for *Re* = 100 and *Ra* = 1.47 × 10^4^.

The numerical benchmark problem is the square chamber with the moving top wall, which is saturated with a Cu-H_2_O nanosuspension. The vertical borders are kept at fixed temperatures and the left border is warmer than the right one. Furthermore, the horizontal walls are insulated. The isotherms acquired by the current code have been compared with those obtained by [23]. As can be seen in Figure 3, for *Re* = 100 and *Ra* = 1.47 × 10^4^, the agreement is good. Figure 4 shows the average *Nu* on the upper boundary in comparison with [23]. It is shown that the results are consistent with the aforementioned study.

### 4.3. Obtained Results and Analysis

**Figure 5:**
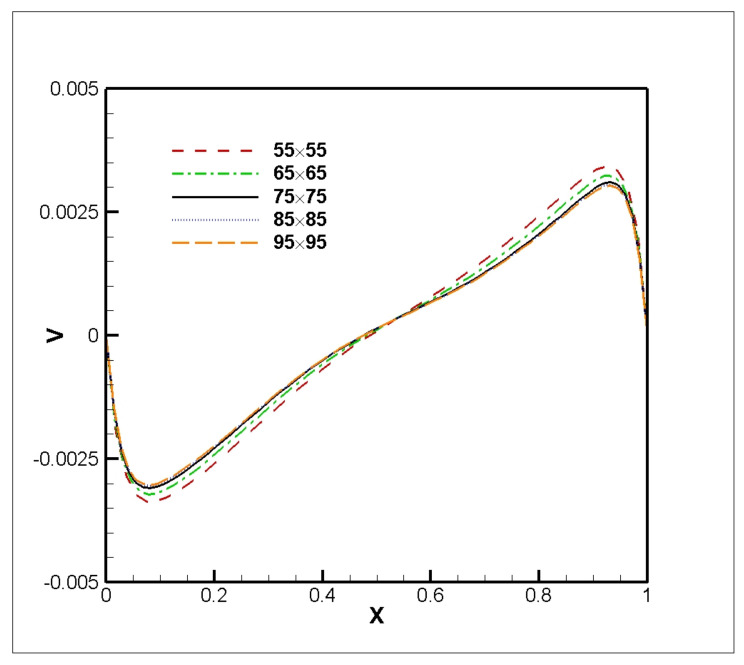
Profiles of vertical velocity *V* versus *X*-axis at *Y* = 0.5 for *Gr* = 100, *Ri* = 0.01, and $\phi_{{\mathrm{Al}}_{2}O_{3}}=0.05,\;\phi_{\mathrm{Cu}}=0.0$.

Here, the fourth-order computational scheme is applied for simulation of the heat and flow in a chamber saturated with an Al_2_O_3_-Cu-H_2_O hybrid nanosuspension for different values of *Ha*, ϕ, and *Ri*. For all simulations, *Gr* = 100 was considered. First, a grid independent study was conducted using the fourth-order compact technique, and the results are shown in Figure 5. The distribution of vertical velocity *V* at *Y* = 0.5 shows that the curves overlap for 75 × 75 and more values. Hence, because of the computational cost, all the simulations were performed on a 75 × 75 grid size.

The average Nusselt number on a hot wall was used as a sensitivity measure of the accuracy of the solution. Table 2 shows the effect of grid quality on the accuracy of the results. The percentage of error confirms that the grid of 75 × 75 elements is appropriate for the simulation. Note that the percentage of error was calculated based on the difference between present and pervious values of the Nusselt number.

**Figure 6:**
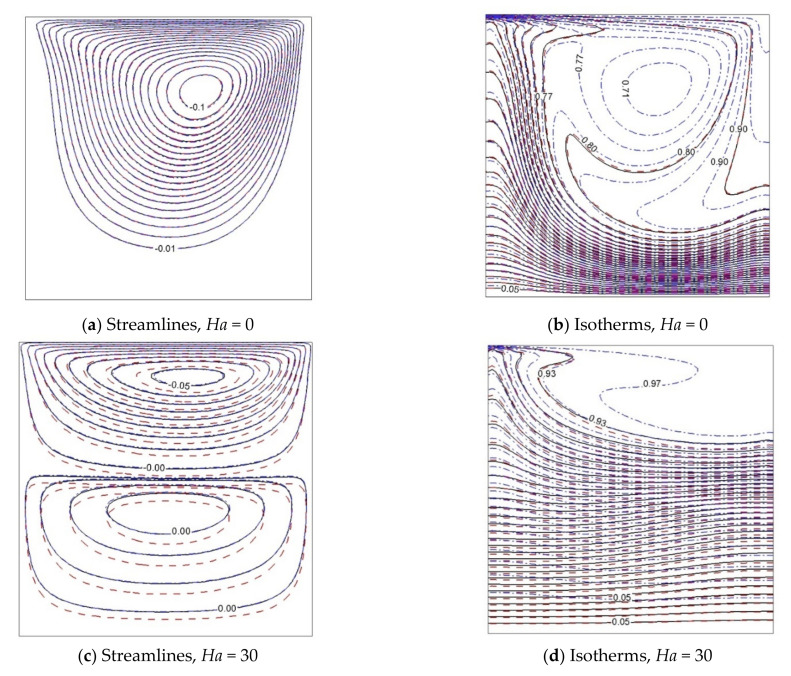

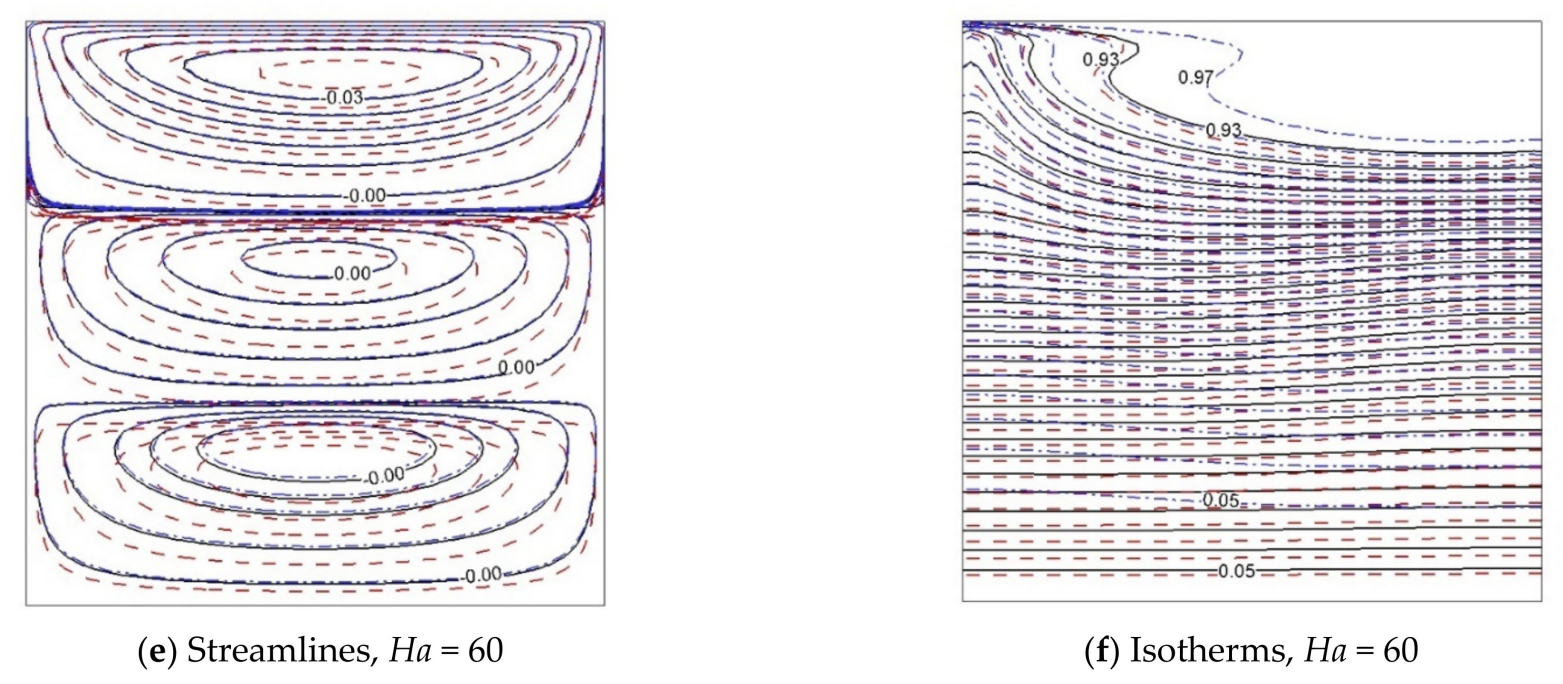
Isolines of ψ and θ for *Gr* = 100, *Ri* = 0.01, and various Hartmann numbers: φ = 0 ––––, $\phi_{{\mathrm{Al}}_{2}O_{3}}=0.05,\;\phi_{\mathrm{Cu}}=0.0$ ------, $\phi_{{\mathrm{Al}}_{2}O_{3}}=0.045,\;\phi_{\mathrm{Cu}}=0.005$ – ⋅ – ⋅ –.

**Figure 7:**
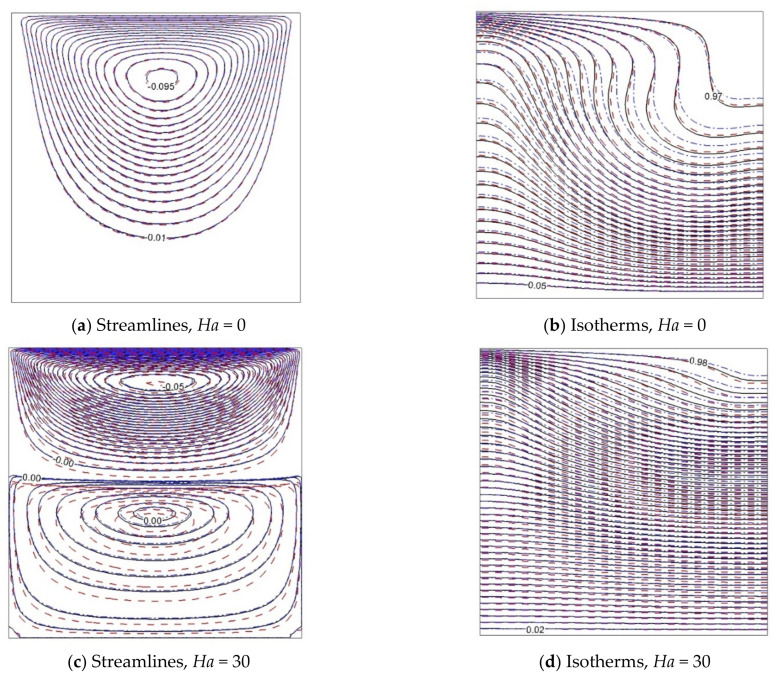

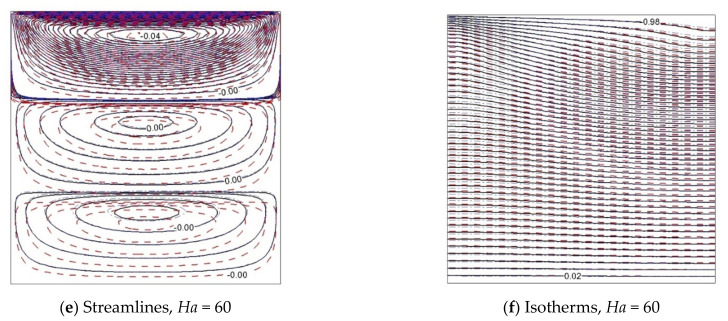
Isolines of ψ and θ for *Gr* = 100, *Ri* = 1, and various Hartmann numbers: φ = 0 ––––, $\phi_{{\mathrm{Al}}_{2}O_{3}}=0.05,\;\phi_{\mathrm{Cu}}=0.0$ ------, $\phi_{{\mathrm{Al}}_{2}O_{3}}=0.045,\;\phi_{\mathrm{Cu}}=0.005$ – ⋅ – ⋅ –.

The effect of adding Al_2_O_3_-Cu and Al_2_O_3_ nanoparticles to the streamlines and isotherms with *Ri* = 0.01 and *Ri* = 1 is shown in Figure 6 and Figure 7. In these figures, the influence of the presence of the magnetic force is investigated. Generally, it can be said that the temperature lines tend to be parallel to magnetic influence. The primary vortex at *Ha* = 0 is divided into two and three vortices when the magnetic force is applied, because the kinetic energy of the fluid decreases with enhancement of the magnetic field. Indeed, the use of hybrid Al_2_O_3_-Cu nanoparticles causes a deformation of temperature lines. In all cases, the streamlines in the hybrid nanofluid and pure fluid stay close together. However, utilizing Al_2_O_3_ nanoparticles dislocates the streamlines under magnetic impact.

**Figure 8:**
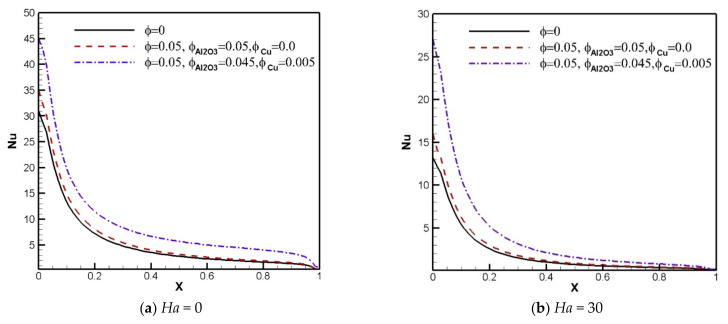

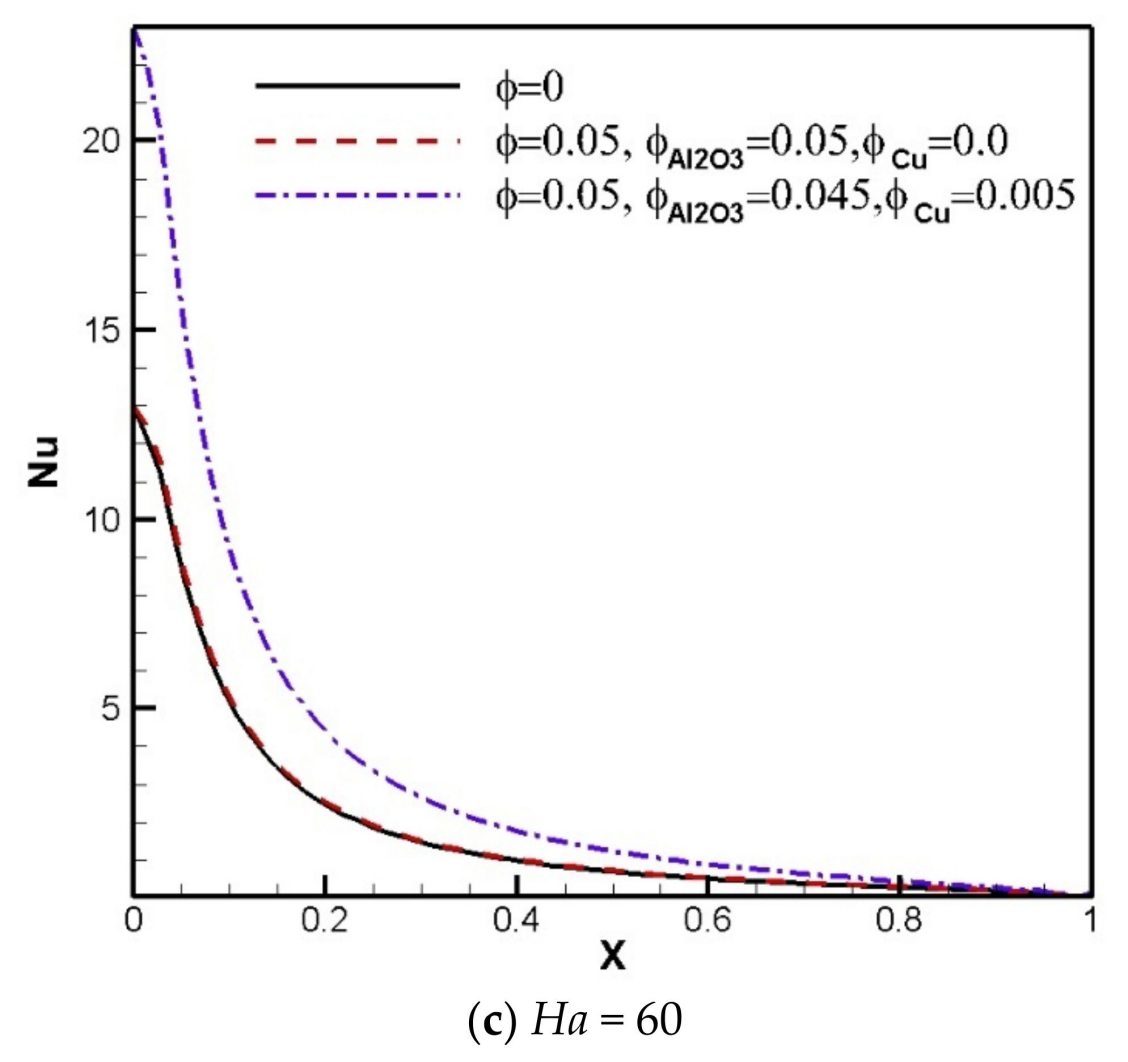
Local Nusselt number profiles versus *X*-axis for *Ri* = 0.01 and various Hartmann numbers and solid volume fractions.

**Figure 9:**
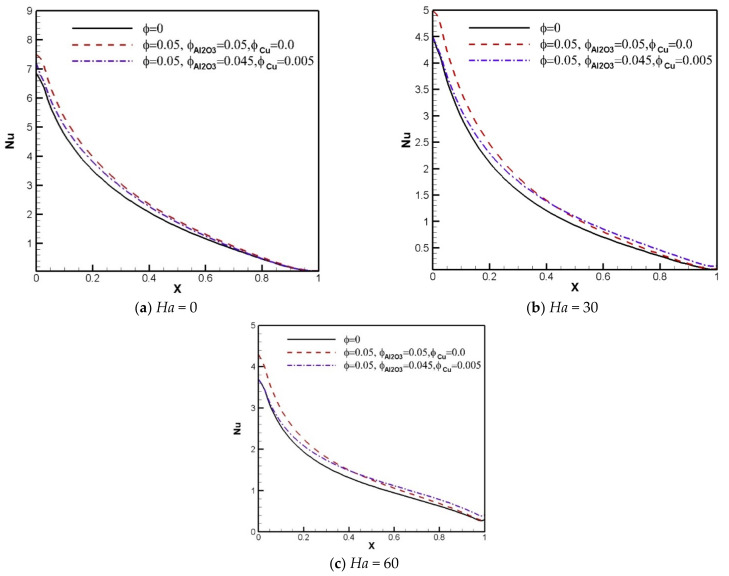
Local Nusselt number profiles versus *X*-axis for *Ri* = 1 and various Hartmann numbers and solid volume fractions.

The effect of the existence of nanoparticles causes changes in the thermal conductivity of the fluid and the strength of the energy transference. Local *Nu* profiles along the heated border in Figure 8 and Figure 9 show the mentioned effects. Generally, the local *Nu* diminishes with increasing *Ha* and *Ri*. The results show that at the lower Richardson number, applying the Al_2_O_3_-Cu hybrid nanoparticles raises *Nu* in comparison with the Al_2_O_3_-H_2_O nanosuspension and pure fluid. At the higher Richardson number, the local Nusselt number curve of the hybrid nanofluid near the left wall remains roughly unchanged in comparison with the pure fluid, but it is higher than the pure fluid and Al_2_O_3_-water nanofluid close to the right wall.

**Figure 10:**
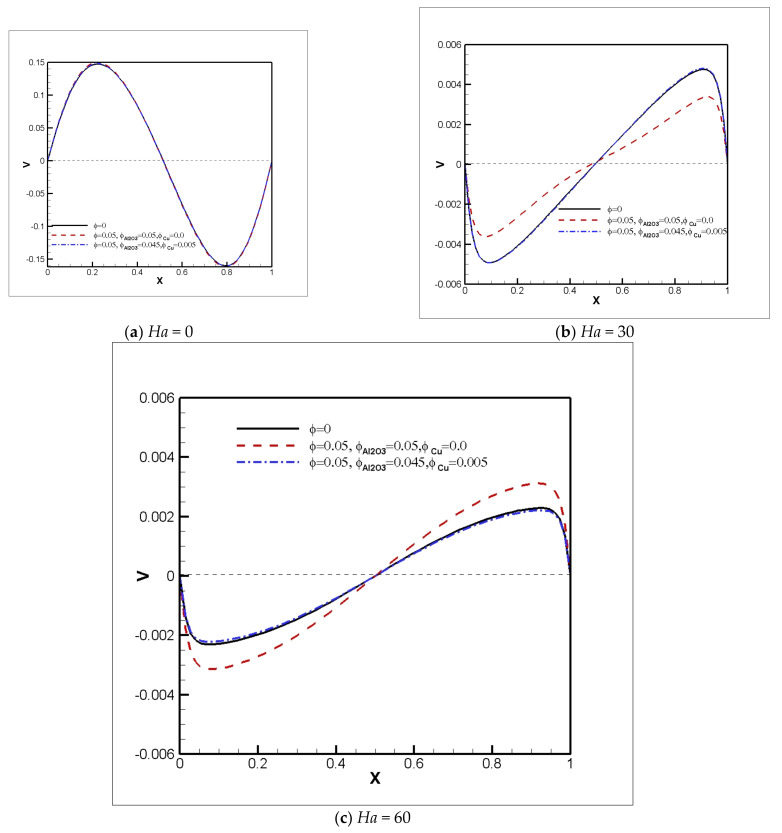
Vertical velocity profiles versus *X*-axis at *Y* = 0.5 for *Ri* = 1 and various Hartmann numbers and solid volume fractions.

The dynamics of flow influenced by the magnetic force and hybrid solid volume fraction are displayed in Figure 10. Figure 10 shows vertical velocity profiles versus *X*-axis at *Y* = 0.5 for different *Ha* and ϕ. Figure 10 shows that as *Ha* increases, the vertical velocity decreases. It is obvious that magnetic influence makes the nanoparticles effective in the dynamics of flow in the cavity. In the vertical velocity profile, weak vortices in the center of the field are displaced by the presence of the Al_2_O_3_-H_2_O nanofluid and the magnetic field. However, by adding Cu nanoparticles, due to the fact that their electrical conductivity is high, they cause the vortices to return to the position of the vortices in the pure fluid, as shown in Figure 5 and Figure 6. In addition, adding a higher percentage of Cu nanoparticles does not cause much change in the results.

## 5. Conclusions

In this paper, the mixed convective motion in a lid-driven heated chamber saturated with an Al_2_O_3_-Cu-H_2_O hybrid nanosuspension and affected by the horizontal magnetic force was numerically simulated. The vorticity-stream function statement was calculated using a high-order compact technique. The results were validated by the available numerical simulations and experimental data. The fluid flow properties and heat convection for various Hartmann numbers (*Ha* = 0–60) and hybrid nanoparticle volume fractions (ϕ = 0–0.05) for *Ri* = 0.01 and *Ri* = 1 were obtained and the following conclusions were reached.

− The energy transference intensity and consequently the Nusselt number were diminished with increments of *Ha* and *Ri*.
− Isotherm patterns were reshaped with the presence of the hybrid nanoparticles for a lower Richardson number.
− Inclusion of Al_2_O_3_ nanoparticles improved the energy transference performance for all studied *Ri* and *Ha*, but adding Cu nanoparticles to the nanofluid at lower *Ri* was highly effective, and at higher *Ri* there was no significant effect.
− The magnetic field intensified the influence of nano-sized particles on the liquid dynamics.
− According to the results, applying the hybrid nanoparticles did not always enhance the heat transfer rate, which means that the other parameters, such as the Richardson number, can affect the presence of hybrid nanoparticles.

## Figures and Tables

**Table 1:** Chemical attributes of H_2_O, Al_2_O_3_ and Cu [14].

	ρ (kg·m^–3^)	β (K^–1^)	*k* (W·m^–1^·K^–1^)	*c* (J·kg^–1^·K^–1^)	σ (S·m^–1^)
H_2_O	997.1	21 × 10^–5^	0.613	4179	0.05
Al_2_O_3_	3970	0.85 × 10^–5^	25	765	1 × 10^–10^
Cu	8933	1.67 × 10^–5^	400	383	5.96 × 10^7^

**Table 2:** Effect of the mesh size on the average Nusselt number for *Gr* = 100, *Ri* = 1, and $\phi_{{\mathrm{Al}}_{2}O_{3}}=0.05,\;\phi_{\mathrm{Cu}}=0.0$.

Number of Nodes	Average Nusselt Number	Percentage of Error
55 × 55	2.4007	-
65 × 65	2.3601	1.6911
75 × 75	2.3523	0.317
85 × 85	2.3486	0.175
95 × 95	2.3456	0.127

## Data Availability

The data presented in this study are available in this paper.
